# Bethesda Assay for Detecting Inhibitory Anti-ADAMTS13 Antibodies in Immune-Mediated Thrombotic Thrombocytopenic Purpura

**DOI:** 10.1055/s-0038-1672187

**Published:** 2018-09-26

**Authors:** Chiara Vendramin, Mari Thomas, John-Paul Westwood, Marie Scully

**Affiliations:** 1 Department of Haematology, University College London Hospital, London, United Kingdom; 2 Department of Haematology, University College London Hospital, Cardiometabolic Programme NIHR UCLH/UCL BRC, London, United Kingdom

**Keywords:** ADAMTS13 protein, Human plasma, anti-ADAMTS13 inhibitors, Bethesda assay, enzyme-linked immunosorbent assay, thrombotic thrombocytopenic purpura

## Abstract

A diagnosis of thrombotic thrombocytopenic purpura (TTP) is confirmed by a severe deficiency (<10%) of a disintegrin and metalloproteinase with a thrombospondin type 1 motif, member 13 (ADAMTS13) activity. Autoantibodies to ADAMTS13 can be detected with a simplified enzyme-linked immunosorbent assay (ELISA). An alternative methodology is a Bethesda assay, which has never been formally assessed in TTP. This study aimed to investigate the inhibitory anti-ADAMTS13 antibody assay and determine if the Bethesda assay is advantageous compared with the ELISA, measuring total immunoglobulin G (IgG) antibodies to ADAMTS 13. The Bethesda method determines the neutralizing activity of anti-ADAMTS13 antibodies in pooled normal plasma. We selected six immune-mediated TTP (iTTP) patients with ADAMTS13 activity levels <10% and strong ADAMTS13 inhibitors by 50:50 mixing studies and analyzed anti-ADAMTS13 antibodies using the Bethesda and ELISA assays. ADAMTS13 activity was stable at room temperature, while a time-dependent decrease in activity was detected in assay conditions of 37°C. Adding 5 mM Ca ^2+^ to citrated plasma prevented loss of ADAMTS13 activity with time. There was time dependence to the antibody-mediated inactivation, after 2-hour incubation. Two of the iTTP patients had no detectable ADAMTS13 antibodies by the Bethesda assay, but had high titer of anti-ADAMTS13 antibodies and low ADAMTS13 antigen levels. The Bethesda assay can only detect anti-ADAMTS13 antibodies that functionally inhibit ADAMTS13. The anti-ADAMTS13 IgG ELISA instead allows the rapid identification of total IgG autoantibodies, detecting both inhibitory and noninhibitory antibodies.

## Introduction

Thrombotic thrombocytopenic purpura (TTP) is a critical life-threating disorder. It is a thrombotic microangiopathy clinically characterized by microangiopathic hemolytic anemia and thrombocytopenia, and involves capillary and small vessel platelet aggregates. A diagnosis of TTP is confirmed by a severe deficiency (<10%) of a disintegrin and metalloproteinase with a thrombospondin type 1 motif, member 13 (ADAMTS13) activity before the first plasma exchange. 1 ADAMTS13 is the key regulator of the hemostatic activity of von Willebrand factor (VWF), accomplished by cleavage of a single site within the A2 domain of VWF. 2 Further assays in the diagnostic workup of immune-mediated TTP (iTTP) include identification of anti-ADAMTS13 immunoglobulin G (IgG) autoantibodies. Methodologies used include enzyme-linked immunosorbent assays (ELISAs) and/or functional inhibitor assays based on mixing studies. 1

Antibodies to ADAMTS13 can be demonstrated in almost all cases of iTTP 3 4 associated with ADAMTS13 activity levels <10% reducing circulating functional enzyme levels. Most autoantibodies were thought to be inhibitory and therefore can be detected and titrated in vitro using classical mixing studies. 5 6 Noninhibitory autoantibodies to ADAMTS13 can be detected with a simplified ELISA that allows the rapid identification of autoantibodies, primarily IgG, using recombinant fragments of ADAMTS13. 7 8 Nonneutralizing antibodies could reduce the amount of circulating ADAMTS13 in the plasma by antibody-mediated clearance. 8

A Bethesda-based assay is used to detect the presence of inhibitory antibodies, such as in hemophilia, but has never been formally assessed in TTP. The aim of this study was to analyze the inhibitory anti-ADAMTS13 antibody assay to understand why currently published Bethesda assay protocols in TTP require a 2-hour incubation period, similar to factor VIII antibodies, 9 10 but not normally undertaken for other inhibitory antibodies when reduced coagulation factor levels are detected. We also wanted to determine if the Bethesda assay had an advantage to the ELISA in detecting and monitoring anti-ADAMTS13 antibodies.

## Materials and Methods

### Patient Samples

Acute TTP was defined as ADAMTS13 protease activity <10% (FRETS VWF73 assay; normal range, 64–132 IU/dL) with a detectable anti-ADAMTS13 IgG antibody present (IgG antibody normal range, <6%). Patients' plasma samples were from the initial presentation of six immune-mediated TTP patients with ADAMTS13 activity levels <10% and strong ADAMTS13 inhibitors by 50:50 mixing studies.

### ADAMTS13 Assays

ADAMTS13 activity was measured using the FRETS VWF73 assay 11 and a published ELISA technique for anti-ADAMTS13 IgG antibody quantification. 8 12 Antibodies were confirmed as inhibitory using a 50:50 mixing study with pooled normal plasma (PNP) and activity measured by the FRETS VWF73 assay as described previously. In mixing tests, the addition of PNP should correct the cleaving protease activity by more than 50% toward normal; that is, if the test sample has 0% activity and the PNP has 100%, then a mixing test result of >50% indicates correction (lack of detection of an activity neutralizing inhibitor), and a mixing test result of <50% is non-correction (indicates that an activity neutralizing inhibitor is present). A strong inhibitor was defined as persisting ADAMTS13 activity <10% after attempts at correction with 50:50 mixing studies. ADAMTS13 antigen levels were quantified using a developed in-house ELISA (ADAMTS13 antigen assay; normal range, 74–134%). 13

### Bethesda Assay

We used a Bethesda method, similar to the one used to analyze inhibitory anti–factor VIII antibodies, 9 to determine the neutralizing activity of anti-ADAMTS13 antibodies in PNP reference plasma and patients' plasma samples.

One Bethesda unit is the amount of inhibitor in 1 mL of plasma that will neutralize 50% of the clotting factor activity (residual activity = 50%), and zero Bethesda units represent 100% residual activity. The numbers of Bethesda units are then corrected for test plasma dilution. In our method, we established the range of dilutions to set up an assay (1/1, 1/2, 1/4, 1/8, 1/16). The residual activity must be between 25 and 75% for accurate results. The aim is to obtain three residual activities between 25 and 75% and determine the mean BU/mL. If the undiluted test plasma gives >75% residual activity, the result has been reported as <0.5 BU/mL, which means “no inhibitor detected.” PNP reference plasma was serially diluted (1/1, 1/2, 1/4, 1/8, 1/16) with phosphate buffer saline (PBS) to obtain a residual ADAMTS13 activity between 25 and 75% and the stability of ADAMTS13 activity at 37°C and room temperature was investigated.

The inhibitor titer was calculated from the residual ADAMTS13 activity measured by the FRETS VWF73 assay after mixing equal volumes of patients' plasma with PNP and incubating the mixture for 10 minutes, 30 minutes, 1 hour, 2 hours, and 4 hours at 37°C. PNP was calibrated against the first international standard for ADAMTS13 12/252 with an activity (by FRETS VWF73 assay) of 96 IU/dL. ADAMTS13 activity in the reaction mixture was read from a PNP reference standard, also preincubated for 10 minutes, 30 minutes, 1 hour, 2 hours, and 4 hours at 37°C before analysis. Patients' plasma was serially diluted (1/1, 1/2, 1/4, 1/8, 1/16) with 20 mM Hepes buffer saline (N-2-hydroxyethylpiperazine-N′-2-ethanesulfonic acid) to obtain a residual ADAMTS13 activity between 25 and 75%. Five mM Ca ^2+^ was added to citrated plasma prior to incubation to restore ADAMTS13 stability.

### Statistical Analysis

All patients were included in the statistical analysis with the Student's *t* -test, Mann–Whitney *U* -test was used as appropriate. GraphPad Prism 6 (GraphPad Software Inc., La Jolla, CA) was used for all statistical analyses.

## Results

### Stability of ADAMTS13 Activity in Human Plasma

**Fig. 1:**
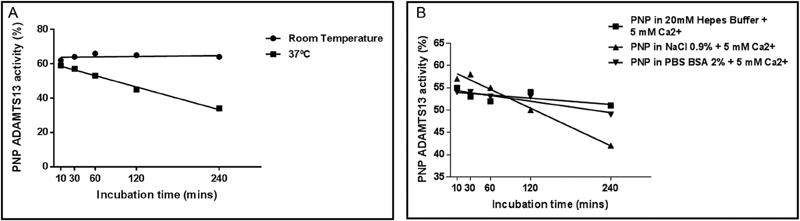
( **A** ) PNP reference plasma ADAMTS13 activity at room temperature and at 37°C. PNP reference plasma was diluted with PBS. A time-dependent decrease of ADAMTS13 activity in PNP reference plasma was observed at 37°C after 60 minutes, while ADAMTS13 proved stable at room temperature. ( **B** ) ADAMTS13 activity of PNP reference plasma. PNP reference plasma was diluted with different solutions, respectively, in 20 mM Hepes buffer saline/sodium chloride 0.9% (NaCl 0.9%), pH 5.7/bovine serum albumin 2% in PBS (PBS BSA 2%) with addition of 5 mM calcium at 37°C. ADAMTS13, a disintegrin and metalloproteinase with a thrombospondin type 1 motif, member 13; PBS, phosphate buffer saline; PNP, pooled normal plasma.

A time-dependent decrease of ADAMTS13 activity in PNP reference plasma was observed at 37°C after 60 minutes, while ADAMTS13 proved stable at room temperature ( Fig. 1A ). We hypothesized that the time-dependent decrease of ADAMTS13 activity in PNP reference plasma observed at 37°C could have been caused by proteolytic degradation; therefore, we incubated samples for 5 to 10 minutes, respectively, with 300 mg/L dabigatran, or 1 mM Pefabloc, or 1 g/L tranexamic acid, before the following incubation step at 37°C. There was no effect on the rate of decline in ADAMTS13. We showed that addition of 5 mM Ca ^2+^ to citrated plasma prior to incubation step at 37°C prevented the decrease of ADAMTS13 activity ( Fig. 1B ). Thereafter, PNP reference plasma was serially diluted (1/1, 1/2, 1/4, 1/8, 1/16) with different solutions (20 mM Hepes buffer saline/sodium chloride—NaCl 0.9%, pH 5.7/bovine serum albumin - BSA 2%), with Hepes proving to be the preferable buffer ( Fig. 1B ).

### Time Dependence of Bethesda Assay

There was time dependence to the antibody-mediated inactivation, after 2 hours of incubation ( Table 1 ). The mean difference between 2- and 4-hour incubation medians was 0.2950 hours (2 hours median, 1.065; 4 hours median, 1.360; 95% confidence interval [CI], −6.370 to 9.070; *p* = 0.9394).

**Table 1:** Analysis of anti-ADAMTS13 antibodies with Bethesda assay and ELISA in six patients with immune-mediated TTP

Patient no.	Incubation time	BU/mL	ADAMTS13: IgG (in-house NR <6%)
1	10′	10.90	
1	30′	10.29	
1	60′	9.78	
1	120′	11.23	95
1	240′	12.02	
2	10′	5.19	
2	30′	4.83	
2	60′	5.56	
2	120′	6.87	98
2	240′	9.57	
3	10′	<0.5	
3	30′	0.79	
3	60′	<0.5	
3	120′	0.83	89
3	240′	1.48	
4	10′	0.50	
4	30′	0.56	
4	60′	0.59	
4	120′	0.87	69
4	240′	1.24	
5	10′	<0.5	
5	30′	<0.5	
5	60′	<0.5	
5	120′	1.26	16
5	240′	<0.5	
6	10′	<0.5	
6	30′	<0.5	
6	60′	<0.5	
6	120′	<0.5	81
6	240′	<0.5	

Abbreviations: ADAMTS13, a disintegrin and metalloproteinase with a thrombospondin type 1 motif, member 13; ELISA, enzyme-linked immunosorbent assay; IgG, immunoglobulin G; NR, normal range; TTP, thrombotic thrombocytopenic purpura.

### Analysis of Patient Anti-ADAMTS13 Response

We selected six iTTP patients with ADAMTS13 activity levels <10% and strong ADAMTS13 inhibitors by 50:50 mixing studies. Two patients were negative for the Bethesda assay, but had a high titer of anti-ADAMTS13 IgG antibodies ( Table 1 ). In four out of six iTTP patients the high titer of anti-ADAMTS13 IgG was confirmed with the Bethesda assay, showing high titer of inhibitory antibodies after 2- and 4-hour incubation period.

Moreover, we correlated the anti-ADAMTS13 antibodies with the results of the other ADAMTS13 parameters: 50:50 mixing study and ADAMTS13 antigen. All the results are summarized in Table 2 . Patients 1, 2, and 5 showed a good correlation between anti-ADAMTS13 antibodies detected with the Bethesda assay and the ELISA, suggesting the antibodies were inhibitory in nature. Conversely, Patients 3 and 4 had high titer levels of anti-ADAMTS13 IgG (89 and 69%, respectively) and low ADAMTS13 antigen levels, but they showed a low titer of inhibitory anti-ADAMTS13 antibodies by the Bethesda method. This would be in keeping with antibody-mediated increased clearance of ADAMTS13. Patient 6 did not show any inhibitory anti-ADAMTS13 antibodies from Bethesda assay, but he had a high titer of anti-ADAMTS13 IgG 81% and a strong ADAMTS13 inhibitor with a 50:50 mixing study less than 5%.

**Table 2:** ADAMTS13-related variables in six patients with immune-mediated TTP

Patient no.	ADAMTS13: activity (FRETS NR 60–123 IU/dL)	ADAMTS13: IgG (in-house NR <6%)	ADAMTS13: Ag (in-house NR 74–134%)	ADAMTS13: inhibitor mixing test (50/50)	BU/mL (NR <0.5)
1	<10	95	10.0	<5	10.84
2	<10	98	15.0	<5	6.40
3	<10	89	1.0	13	1.15
4	<10	69	1.8	<5	0.75
5	<10	16	21.5	36	1.26
6	<10	81	19.3	<5	<0.5

Abbreviations: ADAMTS13, a disintegrin and metalloproteinase with a thrombospondin type 1 motif, member 13; IgG, immunoglobulin G; NR, normal range; TTP, thrombotic thrombocytopenic purpura.

## Discussion

This study illustrates several important aspects concerning the development of a Bethesda assay for detecting inhibitory anti-ADAMTS13 antibodies. ADAMTS13 proved stable at room temperature irrespective of the presence of citrate, while a time-dependent decrease in activity was detected at 37°C in the presence of citrate. A decrease of ADAMTS13 activity of about one-third after 2 hours of incubation of PNP at 37°C has been reported previously. 14 In that study, the hypothesis was that a preincubation temperature of 37°C seemed to influence the enzyme stability when a fluorescence detection–based assay was performed on a plasma matrix 14 and it was suggested to incubate the patient plasma and PNP at 25°C, where ADAMTS13 recovery was maintained. 14 The proteolytic cleavage/inactivation of ADAMTS13 by thrombin, 15 FXa, and plasmin 15 has been described. We tried to inhibit the decrease of ADAMTS13 activity in PNP reference plasma with a direct thrombin inhibitor (dabigatran), with an antifibrinolytic that competitively inhibits the activation of plasminogen to plasmin (tranexamic acid) and with a specific inhibitor of serine proteases (Pefabloc). Addition of these protease inhibitors did not prevent the decline of ADAMTS13 activity, suggesting it was not due to proteolytic degradation. The catalytic domain of ADAMTS13 has different binding sites for metallic cations, one Zn ^2+^ binding site and up to three calcium ion–binding sites. Chelating agents inactivate zinc and calcium ions and therefore influence ADAMTS13 activity. 2 Citrated human plasma serves as the standard source for testing ADAMTS13 and the chelator citrate prevents activation of the coagulation cascade and renders ADAMTS13 inactive. 2 Citrate acts by removing calcium from blood. Unlike EDTA, its mechanism of action is reversible; so, calcium can be added back to study coagulation under controlled conditions. 15 Therefore, 5 mM Ca ^2+^ was added to citrated plasma prior to incubation to restore ADAMTS13 stability.

We also investigated whether there was a rationale for the 2-hour incubation period seen in published Bethesda assay protocols for detecting inhibitory anti-ADAMTS13 antibodies. We used several dilutions of PNP reference plasma (1/1, 1/2, 1/4, 1/8, 1/16) and different solutions including Hepes buffer saline, sodium chloride, and bovine serum albumin. Hepes buffer demonstrated the best assay characteristics. We confirmed at least 2-hour incubation period and, not immediate incubation, is the required time for detecting inhibitory anti-ADAMTS13 antibodies. We concluded that the 2-hour incubation period allows the maximum interaction between the antibody and the enzyme.

Finally, we analyzed the correlation of the anti-ADAMTS13 antibodies with other ADAMTS13 parameters, 50:50 mixing study and ADAMTS13 antigen. Only three of our series had a significantly raised inhibitor by Bethesda assay, with a corresponding inhibitor in mixing test. Two cases had a very low inhibitor by Bethesda, despite a positive mixing test. The very low antigen levels suggest that antibodies may be clearing ADAMTS13. In one case, the Bethesda was negative, mixing tests positive, and ADAMTS13 antigen levels not severely reduced. This result suggests the presence of an inhibitor in this case, but does not appear to have been confirmed using the Bethesda assay. A possible explanation is that our data can indicate an early stage of TTP during which we have just a positive mixing test, while the Bethesda assay is still negative and the ADAMTS13 antigen is not completely cleared. Mixing studies are a useful screening test for an inhibitor without being specific as the Bethesda assays; therefore, they may be positive, whereas the Bethesda assays are negative. These data confirm the presence of the nonneutralizing antibodies as described by Scheiflinger and colleagues 8 and add a considerable impact on the understanding of the pathophysiology of TTP by the inclusion of the ADAMTS13 antigen and mixing test results.

When we compared the Bethesda assay and the ELISA for detecting anti-ADAMTS13 antibodies, some of the results were negative for the Bethesda assay, despite a high titer of anti-ADAMTS13 antibodies. Thus, we confirmed that inhibition is not necessarily the primary effect of the antibodies. A previous study showed that in patients with acute acquired thrombotic microangiopathies associated with severe ADAMTS13 deficiency, autoantibodies are detected more frequently by ELISA than by inhibitor assay. 3 Moreover, the Bethesda assay can detect just anti-ADAMTS13 antibodies that functionally inhibit ADAMTS13, which represents only a part of the complex pathophysiology of the TTP. The use of the FRETS-VWF73 assay to determine the residual activity might in part be an explanation. The FRETS peptide is short and only those antibodies that interfere with the recognition and cleavage of the small VWF substrate contribute to the patient's final inhibitory titer. 16 The ELISA has a better utility and detects total anti-ADAMTS13 antibodies giving more information on the mechanisms involved in causing TTP. Although the number of patients was limited, this study clearly showed the higher clinical utility of anti-ADAMTS13 IgG ELISA assays, which detect both neutralizing and nonneutralizing antibodies, as opposed to the Bethesda assay, which may be negative, despite an immune-mediated mechanism.

In summary, we have described some important assay conditions related to the analysis of antibodies to ADAMTS13 in a Bethesda type assay. Loss of ADAMTS13 activity over time at 37°C can be prevented by addition of calcium and Hepes buffer appeared to be the preferable buffer. Two-hour incubation is sufficient to detect time-dependent antibodies. The Bethesda assay did not detect antibodies in all our cases. Finally, a panel of assays is recommended to fully characterize the pathophysiology of TTP; the anti-ADAMTS13 IgG ELISA may be used as first-line assay, as it would detect anti-ADAMTS13 antibodies in most patients.
